# Multicentre biomarker cohort study on the efficacy of nivolumab treatment for gastric cancer

**DOI:** 10.1038/s41416-020-0975-7

**Published:** 2020-07-03

**Authors:** Takaomi Hagi, Yukinori Kurokawa, Ryohei Kawabata, Takeshi Omori, Jin Matsuyama, Kazumasa Fujitani, Motohiro Hirao, Yusuke Akamaru, Tsuyoshi Takahashi, Makoto Yamasaki, Taroh Satoh, Hidetoshi Eguchi, Yuichiro Doki

**Affiliations:** 1 Department of Gastroenterological Surgery, Osaka University Graduate School of Medicine, Osaka, Japan; 2 Department of Surgery, Osaka Rosai Hospital, Osaka, Japan; 3 Department of Gastroenterological Surgery, Osaka International Cancer Institute, Osaka, Japan; 4 Department of Gastroenterological Surgery, Higashiosaka City Medical Center, Osaka, Japan; 5 Department of Surgery, Osaka General Medical Center, Osaka, Japan; 6 Department of Surgery, National Hospital Organization Osaka National Hospital, Osaka, Japan; 7 Department of Gastroenterological Surgery, Ikeda City Hospital, Osaka, Japan; 8 Department of Frontier Science for Cancer and Chemotherapy, Osaka University Graduate School of Medicine, Osaka, Japan

**Keywords:** Predictive markers, Predictive markers

## Abstract

**Background:**

Predictive factors of nivolumab treatment response in patients with gastric cancer (GC) remain unclear.

**Methods:**

In this retrospective cohort study, tissue specimens of patients with unresectable or recurrent GC and prior or scheduled treatment with nivolumab as third-line or higher therapy between September 2017 and February 2019 were collected from 23 institutions. The tumour-positive score (TPS) and combined positive score (CPS) of PD-L1 expression and mismatch repair (MMR) were analysed by immunohistochemistry. Associations between clinicopathological factors and tumour-response rate, hyperprogressive disease (HPD) rate and survival were assessed.

**Results:**

Of 200 eligible patients, 143 had measurable lesions. The response and HPD rates were 17.5% and 22.1%, respectively. The response rate was significantly higher in patients with performance status (PS) 0–1 (*P* = 0.026), non-peritoneal metastasis (*P* = 0.021), PD-L1 TPS ≥ 1 (*P* = 0.012), CPS ≥ 5 (*P* = 0.007) or ≥ 10 (*P* < 0.001) or MMR deficiency (*P* < 0.001). The HPD rate was significantly higher in patients with PS 2–3 (*P* = 0.026), liver metastasis (*P* < 0.001) and CPS < 10 (*P* = 0.048). Multivariate analysis revealed that CPS (*P* = 0.001) and MMR (*P* = 0.002) were independent prognostic factors of progression-free survival, as well as liver metastasis (*P* < 0.001), peritoneal metastasis (*P* = 0.004) and CRP (*P* < 0.001).

**Conclusions:**

PD-L1 CPS and MMR could be useful biomarkers for nivolumab treatment efficacy in GC.

**Clinical trial registration:**

UMIN000032164.

## Background

Gastric cancer (GC) is one of the most common types of digestive cancer and is the third leading cause of death worldwide.^1^ Despite recent advances in cytotoxic chemotherapy, the prognosis of unresectable or recurrent GC remains poor, and more effective treatments are needed to improve survival.^2–6^ Immunotherapy is a new paradigm for the treatment of GC, and targeting the programmed death-1 (PD-1) pathway is a promising therapeutic option.^7^

Recently, nivolumab, a humanised IgG4 monoclonal antibody inhibitor of PD-1, demonstrated survival benefit in patients with advanced GC in a Phase 3 trial (ATTRACTION-2).^8^ Moreover, pembrolizumab, another PD-1 monoclonal antibody, also showed antitumour effects in patients with advanced GC in Phase 2 and 3 trials.^9,10^ However, in these trials, approximately 60–70% of patients who were treated with PD-1 monoclonal antibody for GC exhibited no response. In addition, several studies revealed that paradoxical accelerated tumour progression, known as hyperprogressive disease (HPD), was observed in some patients after initiation of therapy with antibodies against PD-1 or programmed death-ligand 1 (PD-L1).^11–13^ Taken together, it is necessary to identify precise predictive biomarkers to determine which patients will exhibit positive or negative effects following PD-1 blockade.

The exploratory subgroup analysis of ATTRACTION-2 indicated that nivolumab improved overall survival (OS), regardless of PD-L1 expression on tumour cells. On the other hand, the degree of PD-L1 expression evaluated by the combined positive score (CPS), which includes both PD-L1-positive tumour and immune cells, might be related to the effect of treatment with pembrolizumab.^10,14^ In addition, microsatellite instability (MSI)-high, i.e., mismatch repair (MMR) deficiency, was suggested to be a predictive factor for response to PD-1 blockade.^15,16^ A small study showed that several clinicopathological and molecular factors, such as poor performance status (PS), PD-L1 positivity in tumour cells (not in immune cells) and MMR deficiency, were associated with the response to nivolumab in patients with GC.^17^ However, predictive factors of nivolumab response in GC have not been evaluated in a larger cohort. In this multicentre cohort study, we analysed real-world data regarding nivolumab treatment in a relatively large number of patients. We also investigated predictive factors of nivolumab responders and HPD patients by examining the association between clinicopathological characteristics and response to nivolumab in patients with GC.

## Methods

### Patients

This cohort study included patients with unresectable or recurrent GC who had been treated or were scheduled to be treated with nivolumab as third-line or higher therapy between September 2017 and February 2019 at any of the 23 institutions of the Clinical Study Group of Osaka University, Upper Gastrointestinal Surgery Group. The eligibility criteria were as follows: histologically diagnosed with adenocarcinoma of the stomach or gastroesophageal junction, refractory or intolerant to two or more previous chemotherapy regimens, previous or anticipated treatment with nivolumab alone and 20 years of age or older. Patients who were previously treated with any immune-checkpoint inhibitor other than nivolumab were ineligible. Patients who had synchronous or metachronous (within 5 years) malignancy other than carcinoma in situ or mucosal carcinoma at the start of nivolumab treatment were excluded. Patients gave written informed consent before enrolment. Only for patients who were dead or lost to follow-up, informed consent was not required. The study was approved by the institutional review boards of all participating institutions. Names of the ethics committees of all institutions and the reference numbers of this study are shown in Supplementary Table. This study is registered with UMIN Clinical Trials Registry, number UMIN000032164.

### Immunohistochemistry and evaluations

Tumour tissue samples were obtained from either endoscopic biopsy at diagnosis or surgically resected specimens. Specimens were fixated by 10% neutral buffered formalin for 24–72 h in most of the cases and embedded in paraffin at each institution. Tissue samples were shipped to the study centre for central pathological review.

Immunohistochemical analysis was conducted using 4-µm-thick tissue sections. The primary antibody for PD-L1 immunohistochemistry was VENTANA PD-L1 (SP263) Rabbit Monoclonal Primary Antibody (Ventana Medical Systems, Tucson, AZ, USA). Immunostaining for PD-L1 was performed using a BenchMark GX IHC/ISH system with VENTANA OptiView DAB IHC Detection Kit (Ventana Medical Systems). Positive PD-L1 expression was designated when the membrane of tumour cells, lymphocytes and macrophages was stained in comparison with that of positive control (placenta). For the evaluation of PD-L1 expression, TPS was defined as the number of PD-L1-staining tumour cells divided by the total number of viable tumour cells multiplied by 100. CPS was defined as the number of PD-L1-staining cells (tumour cells, lymphocytes and macrophages) divided by the total number of viable tumour cells multiplied by 100. Representative immunohistochemical staining according to PD-L1 TPS and CPS is shown in Supplementary Fig. S1.

MMR status was evaluated by immunohistochemistry of mutL homologue 1 (MLH1), mutS homologue 2 (MSH2), mutS homologue 6 (MSH6) and postmeiotic segregation increased 2 (PMS2). Primary antibodies were monoclonal antibodies for anti-MLH1 (ES05, mouse) and anti-MSH2 (79H11, mouse) from Leica Biosystems (Nussloch, Germany), and anti-MSH6 (EP49, rabbit) and anti-PMS2 (EP51, rabbit) from Agilent Technologies (Santa Clara, CA, USA). Immunostaining for MLH1, MSH2, MSH6 and PMS2 was performed as previously described.^18^ For determining expression of each MMR protein, positive expression was defined as the presence of nuclear staining of tumour cells without regard to the proportion or intensity, and the nuclear staining of each cancer cell was evaluated in comparison with that of normal epithelium and the positive control (tonsil). When nuclear staining was not present in any of the tumour cells, but observed in normal epithelium and the positive control, it was considered as negative. Tumours without expression of MLH1, MSH2, MSH6 or PMS2 were considered to be MMR-deficient, while tumours with MLH1, MSH2, MSH6 and PMS2 expression were considered to be MMR-proficient. Representative immunohistochemical staining with MLH1, MSH2, MSH6 and PMS2 is shown in Supplementary Fig. S2. Immunohistochemistry analysis was conducted while blind to the clinical data under the supervision of two pathologists of the department of Pathology, Osaka University Hospital.

### Evaluations of tumour response

Although the follow-up schedule was not specified in this study, the efficacy evaluation was conducted every 6–8 weeks in most of the patients (91.0%). Tumour response was assessed according to Response Evaluation Criteria in Solid Tumors version 1.1 (RECIST v1.1). A minimum interval of 6 weeks between two measurements was required for determination of complete response (CR), partial response (PR) and stable disease (SD). Non-evaluable (NE) patients were regarded as non-responders. Response rate was assessed only in patients with measurable lesions. Response rate was defined as the proportion of patients with a best overall response of CR or PR; both groups were considered to be responders.

HPD was assessed in patients with measurable lesions who had undergone a CT scan during previous chemotherapy (pre-baseline CT) and also after starting nivolumab treatment (post CT). HPD was defined using tumour growth kinetics (TGK) as previously described.^11,14^ Briefly, *S*_PRE_, *S*_0_ and *S*_POST_ represented the sum of the longest diameters of the target lesions according to RECIST v1.1 and at pre-baseline, baseline and post CT, respectively. *T*_PRE_, *T*_0_ and *T*_POST_ represented the pre-baseline, baseline and post CT time points, respectively. TGK_PRE_ was defined as the difference between pre-baseline and baseline CT in the sum of the longest diameters of the target lesions per unit of time: (*S*_0_ − *S*_PRE_)/(*T*_0_ − *T*_PRE_). Similarly, TGK_POST_ was described as (*S*_POST_ − *S*_0_)/(*T*_POST_ − *T*_0_). The TGK ratio was defined as TGK_POST_/TGK_PRE_. HPD was defined as a TGK ratio ≥ 2 and (*S*_POST_/*S*_0_ − 1) >0.5, as previously described.^11–13^

### Statistical analysis

The relationships between clinicopathological characteristics, including PD-L1 or MMR status of tissue samples and tumour-response status, were analysed using the chi-square test for categorical variables. Progression-free survival (PFS) was defined as the interval from the date of the first administration of nivolumab to the date of disease progression or death from any cause. OS was defined as the interval from the date of the first administration of nivolumab to the date of death due to any cause. Survival rates were estimated using the Kaplan–Meier method and were compared with the log-rank test. The prognostic variables that were significantly associated with PFS in the univariate analyses were further assessed in multivariate Cox proportional hazard model analyses. *P* < 0.05 was considered to indicate statistical significance. Analyses were performed with SPSS software, version 22.0 (IBM Corp., Armonk, NY, USA).

## Results

### Patient characteristics and expression status

**Fig. 1:**
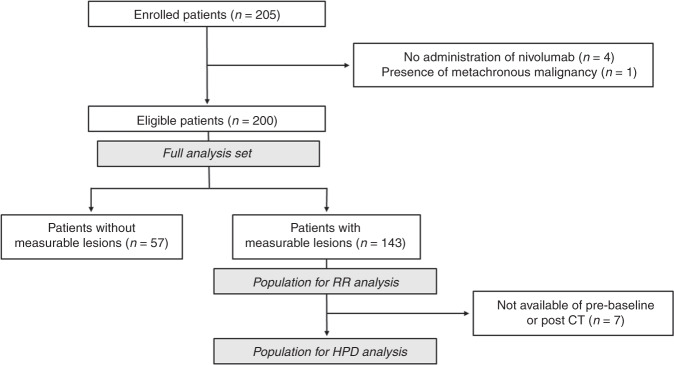
Flow chart of patient eligibility for inclusion in the study. RR response rate, HPD hyperprogressive disease, CT computed tomography.

The patient flow diagram for this study is shown in Fig. 1. Among 205 GC patients who were enrolled in this study, five were ineligible, for the following reasons: four did not receive nivolumab treatment and one demonstrated metachronous malignancy. Of the 200 patients who were eligible for study inclusion, 143 had measurable lesions, while 57 did not, and the proportions of patients with specific PD-L1 expression statuses were as follows: TPS ≥ 1, 25.0%; CPS ≥ 1, 58.5%; CPS ≥ 5, 37.0%; CPS ≥ 10, 19.5%. Of 200 patients, 21 (10.5%) showed negative MLH1 expression, 5 (2.5%) showed negative MSH2 expression, 4 (2.0%) showed negative MSH6 expression and 14 (7.0%) showed negative PMS2 expression. Overall, the proportion of patients who were MMR- deficient was 14.5%.

### Response rates following nivolumab treatment

Among 143 patients with measurable lesions, the best overall response was CR in one patient, PR in 24 patients, SD in 18 patients, progressive disease (PD) in 98 patients and NE in two patients. Thus, the response rate in this study was 17.5% (25/143). The relationships between clinicopathological characteristics and response rate are shown in Table 1. Patients with a PS of 0–1 showed a significantly higher response rate than those with a PS of 2–3 (20.3% vs. 0.0%, *P* = 0.026). Patients with peritoneal metastasis showed a significantly lower response rate than those without peritoneal metastasis (6.7% vs. 22.4%, *P* = 0.021). The other clinical characteristics showed no significant correlation with response rate. Regarding PD-L1 expression, TPS ≥ 1 (*P* = 0.012), CPS ≥ 5 (*P* = 0.007) and CPS ≥ 10 (*P* < 0.001) showed a significant association with response, and patients with CPS ≥ 10 demonstrated a higher response rate (40.6%) than those with TPS ≥ 1 (31.4%) or CPS ≥ 5 (28.8%). Although patients with PD-L1 CPS ≥ 1 showed higher response rate (21.6%) than those with CPS < 1 (10.9%), it was not significant (*P* = 0.10). Meanwhile, patients with MMR deficiency showed a significantly higher response rate than those with MMR proficiency (47.6% vs. 12.3%, *P* < 0.001).

**Table 1:** Clinicopathological characteristics in responders and non-responders.

		Responder (*n* = 25)	Non-responder (*n* = 118)	Response rate (%)	*P* value
Age (years)	<70	8	51	13.6	0.30
	≥70	17	67	20.2	
Sex	Male	19	91	17.3	0.90
	Female	6	27	18.2	
Performance status	0–1	25	98	20.3	0.026
	2–3	0	20	0.0	
BMI (kg/m^2^)	<20	11	57	16.2	0.70
	≥20	14	61	18.7	
Histology^*^	Differentiated	16	77	17.2	0.89
	Undifferentiated	8	41	16.3	
History of gastrectomy	Yes	18	66	21.4	0.14
	No	7	52	11.9	
Number of previous chemotherapy regimens	2	14	66	17.5	0.64
	3	6	36	14.3	
	≥4	5	16	23.8	
Liver metastasis	Yes	7	49	12.5	0.21
	No	18	69	20.7	
Lung metastasis	Yes	2	10	16.7	0.94
	No	23	108	17.6	
Lymph node metastasis	Yes	16	63	20.3	0.33
	No	9	55	14.1	
Peritoneum metastasis	Yes	3	42	6.7	0.021
	No	22	76	22.4	
NLR	<2.4	11	58	15.9	0.64
	≥2.4	14	60	18.9	
CRP (mg/dL)^*^	<1.0	21	78	21.2	0.087
	≥1.0	4	39	9.3	
PD-L1 TPS	<1	14	94	13.0	0.012
	≥1	11	24	31.4	
PD-L1 CPS	<1	6	49	10.9	0.10
	≥1	19	69	21.6	
PD-L1 CPS	<5	10	81	11.0	0.007
	≥5	15	37	28.8	
PD-L1 CPS	<10	12	99	10.8	<0.001
	≥10	13	19	40.6	
MMR	Proficient	15	107	12.3	<0.001
	Deficient	10	11	47.6	

^*^Data not available for one patient.

*BMI* body mass index, *NLR* neutrophil-to-lymphocyte ratio, *CRP* C-reactive protein, *PD-L1* programmed death-ligand 1, *TPS* tumour-positive score, *CPS* combined positive score, *MMR* mismatch repair.

### HPD following nivolumab treatment

Of 143 patients with measurable lesions, seven patients who did not undergo pre-baseline or post CT were excluded from the analysis of HPD. Among 136 patients, 30 (22.1%) satisfied the definition of HPD. The relationships between clinicopathological characteristics and HPD rate are shown in Table 2. Patients with a PS of 2–3 showed a significantly higher HPD rate than those with a PS of 0–1 (43.8% vs. 19.2%, *P* = 0.026). Patients with liver metastasis showed a significantly higher HPD rate than those without liver metastasis (42.6% vs. 8.5%, *P* < 0.001). The other clinical characteristics showed no significant correlation with HPD rate. Considering PD-L1 expression, only CPS ≥ 10 showed a significant correlation with low HPD rate (*P* = 0.048), while the other molecular characteristics showed no correlation.

**Table 2:** Clinicopathological characteristics in patients with and without hyperprogressive disease.

		HPD patients (*n* = 30)	Non-HPD patients (*n* = 106)	HPD rate (%)	*P* value
Age (years)	<70	13	42	23.6	0.72
	≥70	17	64	21.0	
Sex	Male	25	82	23.4	0.48
	Female	5	24	17.2	
Performance status	0–1	23	97	19.2	0.026
	2–3	7	9	43.8	
BMI (kg/m^2^)	<20	11	52	17.5	0.23
	≥20	19	54	26.0	
Histology^*^	Differentiated	20	71	22.0	0.92
	Undifferentiated	10	34	22.7	
History of gastrectomy	Yes	15	67	18.3	0.19
	No	15	39	27.8	
Number of previous chemotherapy regimens	2	18	58	23.7	0.76
	3	7	32	17.9	
	≥4	5	16	23.8	
Liver metastasis	Yes	23	31	42.6	<0.001
	No	7	75	8.5	
Lung metastasis	Yes	2	10	16.7	0.64
	No	28	96	22.6	
Lymph node metastasis	Yes	12	62	16.2	0.073
	No	18	44	29.0	
Peritoneum metastasis	Yes	10	31	24.4	0.67
	No	20	75	21.1	
NLR	<2.4	14	53	20.9	0.75
	≥2.4	16	53	23.2	
CRP (mg/dL)^*^	<1.0	18	80	18.4	0.080
	≥1.0	12	25	32.4	
PD-L1 TPS	<1	24	77	23.8	0.42
	≥1	6	29	17.1	
PD-L1 CPS	<1	14	36	28.0	0.20
	≥1	16	70	18.6	
PD-L1 CPS	<5	22	62	26.2	0.14
	≥5	8	44	15.4	
PD-L1 CPS	<10	27	77	26.0	0.048
	≥10	3	29	9.4	
MMR	Proficient	27	88	23.5	0.35
	Deficient	3	18	14.3	

^*^Data not available for one patient.

*HPD* hyperprogressive disease, *BMI* body mass index, *NLR* neutrophil-to-lymphocyte ratio, *CRP* C-reactive protein, *PD-L1* programmed death-ligand 1, *TPS* tumour-positive score, *CPS* combined positive score, *MMR* mismatch repair.

### Survival

**Fig. 2:**
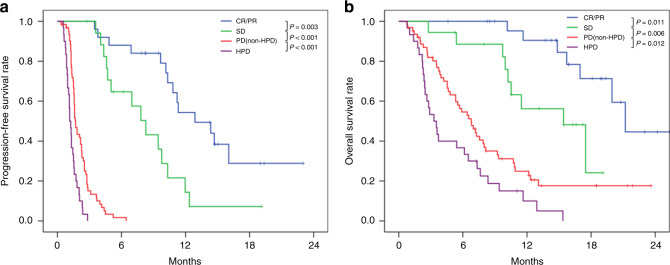
Kaplan–Meier progression-free and overall survivals for 136 patients who had measurable lesions according to response status. **a** Progression-free survival, **b** overall survival in complete response (CR) or partial response (PR) (*n* = 25), stable disease (SD) (*n* = 18), progressive disease (PD) without hyperprogressive disease (HPD) (*n* = 63) and HPD (*n* = 30).

The median follow-up periods of PFS and OS for the censored patients were 8.2 months and 13.7 months, respectively. The median PFS and OS for nivolumab were 2.2 months (95% confidence interval [CI] 1.7–2.7 months) and 7.6 months (95% CI 5.7–9.6 months), respectively. Kaplan–Meier survival curves of PFS and OS in the 136 patients according to response status are shown in Fig. 2. The median PFS for nivolumab in CR/PR, SD, PD (non-HPD) and HPD patients were 12.9, 8.3, 1.7 and 1.2 months, respectively. There were significant differences in PFS between CR/PR and SD (*P* = 0.003), SD and PD (non-HPD) (*P* < 0.001) and PD (non-HPD) and HPD patients (*P* < 0.001). Furthermore, the median OS for nivolumab in CR/PR, SD, PD (non-HPD) and HPD patients were 21.2, 15.4, 6.8 and 3.3 months, respectively. There were significant differences in OS between CR/PR and SD (*P* = 0.011), SD and PD (non-HPD) (*P* = 0.006) and PD (non-HPD) and HPD patients (*P* = 0.012). Of 200 patients, 80 (40.0%) patients received at least one subsequent treatment. The common regimens were irinotecan (23.0%), paclitaxel (10.5%) and ramucirumab (7.5%).

**Fig. 3:**
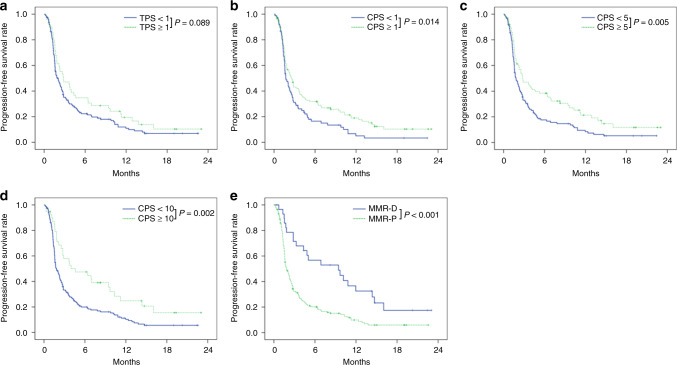
Kaplan–Meier progression-free survival for all 200 patients according to PD-L1 and MMR status. **a** Tumour-positive score (TPS) <1 (*n* = 150) or TPS ≥1 (*n* = 50), **b** combined positive score (CPS) <1 (*n* = 83) or ≥1 (*n* = 117), **c** CPS <5 (*n* = 126) or ≥5 (*n* = 74), **d** CPS <10 (*n* = 161) or ≥10 (*n* = 39) and (**e**) mismatch repair (MMR) deficient (*n* = 29) or proficient (*n* = 171).

The subgroup analyses of PFS for all 200 patients according to tumour PD-L1 and MMR status are shown in Fig. 3. No significant difference in PFS was seen between PD-L1 TPS ≥ 1 and TPS < 1 (*P* = 0.089). On the other hand, patients who were PD-L1 CPS-positive showed significantly better PFS than those who were negative according to any cut-off value: CPS ≥ 1 (*P* = 0.014), CPS ≥ 5 (*P* = 0.005) and CPS ≥ 10 (*P* = 0.002). PFS was significantly better in patients with MMR deficiency than MMR proficiency (*P* < 0.001). Univariate analysis showed that age, PS, history of gastrectomy, liver metastasis, peritoneal metastasis, CRP, PD-L1 CPS for any cut-off value and MMR were significant prognostic factors of PFS (Table 3). A Cox multivariate analysis for PFS with these eight clinicopathological covariables revealed that the presence of liver metastasis, presence of peritoneal metastasis, CRP ≥ 1.0 mg/dL, PD-L1 CPS ≥ 10 and MMR proficiency were independent indicators of poor PFS.

**Table 3:** Univariate and multivariate analyses for progression-free survival.

Variables	Category	Univariate analysis		Multivariate analysis	
		HR (95% CI)	*P* value	HR (95% CI)	*P* value
Age (years)	<70	1.37 (1.01–1.85)	0.044	1.31 (0.96–1.80)	0.093
Sex	Female	1.23 (0.86–1.74)	0.25		
Performance status	2–3	2.08 (1.35–3.21)	0.001	1.48 (0.93–2.36)	0.102
BMI (kg/m^2^)	<20	1.10 (0.81–1.49)	0.54		
Histology^*^	Undifferentiated	1.16 (0.85–1.58)	0.35		
History of gastrectomy	No	1.38 (1.01–1.89)	0.045	1.04 (0.74–1.46)	0.83
Number of previous chemotherapy regimens	<3	1.16 (0.85–1.59)	0.35		
Liver metastasis	Yes	1.55 (1.12–2.14)	0.008	2.01 (1.40–2.89)	<0.001
Lung metastasis	No	1.33 (0.72–2.45)	0.36		
Lymph node metastasis	No	1.12 (0.83–1.51)	0.47		
Peritoneum metastasis	Yes	1.43 (1.06–1.94)	0.021	1.65 (1.17–2.33)	0.004
NLR	≥2.4	1.12 (0.82–1.51)	0.48		
CRP (mg/dL)^*^	≥1.0	2.01 (1.44–2.82)	<0.001	2.00 (1.36–2.93)	<0.001
PD-L1 TPS	<1	1.35 (0.95–1.92)	0.092		
PD-L1 CPS	<1	1.46 (1.07–1.98)	0.016		
PD-L1 CPS	<5	1.57 (1.14–2.17)	0.005		
PD-L1 CPS	<10	1.87 (1.25–2.80)	0.002	1.96 (1.30–2.97)	0.001
MMR	Proficient	2.35 (1.48–3.75)	<0.001	2.12 (1.32–3.39)	0.002

^*^Data not available for one patient.

*HR* hazard ratio, *CI* confidence interval, *BMI* body mass index, *NLR* neutrophil-to-lymphocyte ratio, *CRP* C-reactive protein, *PD-L1* programmed death-ligand 1, *TPS* tumour-positive score, *CPS* combined positive score, *MMR* mismatch repair.

## Discussion

Our multicentre cohort study of 200 patients with GC demonstrated real-world results of nivolumab treatment: the response and HPD rates were 17.5% and 22.1%, respectively. The median OS and PFS in our CR/PR patients were 21.2 and 12.9 months, respectively, although the ATTRACTION-2 trial reported that the median OS for nivolumab in their CR/PR patients was 26.6 months.^19^ Our study revealed that PS, peritoneal metastasis, PD-L1 CPS and MMR were associated with nivolumab response. In addition, PS, liver metastasis and PD-L1 CPS showed significant associations with HPD in nivolumab treatment. Most of these factors showed a distinct influence on PFS, suggesting that they may be predictive factors of nivolumab treatment. Our study also demonstrated that PD-L1 CPS was better than PD-L1 TPS as a biomarker for nivolumab treatment. A large-scale prospective study is warranted to validate our findings.

Previous studies reported that patients with MMR deficiency showed a good response to PD-1 blockade for solid tumours, including GC.^15,16,20^ Therefore, pembrolizumab received approval by the US Food and Drug Administration in May 2017 for the treatment of patients with MSI-high or MMR-deficient solid tumours. Consistent with these results, patients with MMR deficiency had a better response rate and prognosis compared with those with MMR proficiency. Since MSI-high has been reported as a predictive marker of non-response to fluorouracil-based chemotherapy,^21–23^ a new strategy might involve administering PD-1 blockade at an earlier phase or as an adjuvant therapy for those with MMR-deficient GC. Our results also showed an association between PS and response to nivolumab. Although there is no definitive explanation for this finding, patients with poor PS might not be able to tolerate the treatment well enough to achieve a satisfactory response.

A Phase 3 trial of pembrolizumab indicated that PD-L1 CPS may be superior to PD-L1 TPS as a predictive biomarker of response to pembrolizumab in patients with GC.^10^ Mishima et al. reported that PD-L1 TPS ≥ 1, but not PD-L1 CPS ≥ 10, was significantly associated with response,^17^ which was contrary to our results. One reason for this discrepancy may be the clones of anti-PD-L1 antibody that were employed; they used SP142 or SP263, while we used only SP263. Since 28-8 and 22C3, that were often used in clinical studies, showed high concordance with SP263 in terms of PD-L1 expression on tumour cells,^24^ SP142 was reported to be an outlier that detected significantly less PD-L1 expression compared with the other clones.^25–27^ In KEYNOTE062, a Phase 3 trial comparing the efficacy of cytotoxic chemotherapy combined with pembrolizumab vs. only cytotoxic chemotherapy vs. only pembrolizumab as first-line therapy for GC, pembrolizumab demonstrated a meaningful improvement in OS in PD-L1 CPS ≥ 10 subgroups,^28^ which supports our results.

In this study, we defined HPD as a TGK ratio ≥ 2 and > 50% increase in tumour burden. Using a similar definition of HPD as employed in our study, Sasaki et al. and Saâda-Bouzid et al. reported the respective HPD incidences of 21% in patients with GC^11^ and 29% in patients with head and neck squamous cell carcinoma.^13^ On the other hand, Kato et al. reported that only 6% of patients with various cancer types experienced HPD as defined by the combination of our criteria and < 2 months of time-to-treatment failure.^12^ Moreover, 9–14% of patients with other cancer types experienced HPD, and this accounted for three-dimensional tumour growth.^29,30^ These results suggest that standardising the definition of HPD will be necessary in the future. According to our results, poor PS, liver metastasis and PD-L1 CPS < 10 may be predictors of HPD, and these findings are similar to those of a previous study.^11^ PD-1 blockade in patients with poor PS might cause changes in immune status, which might in turn increase the secretion of cytokines and other mediators and thus facilitate immune escape.^31,32^ Liver-induced immune tolerance might also cause paradoxical acceleration of liver metastasis treated by PD-1 blockade.^33,34^ Recent studies suggested that PD-1-positive regulatory T cells amplified by PD-1 blockade promoted HPD.^35^ However, the mechanism of HPD remains unclear. Further studies are needed to identify individuals who might be harmed by PD-1 blockade.

One of the limitations of this study is that it used a retrospective design. However, it was a multicentre cohort study that included 23 institutions, and the data of consecutive patients with GC who were treated with nivolumab were obtained from every institution. Therefore, we believe that selection bias was minimised. On the other hand, since the patient follow-up schedule was not specified in this study due to the retrospective nature, it might have affected PFS outcomes. Second, MMR status was evaluated by immunohistochemistry of MMR proteins, not by MSI testing. Although all procedures of staining were conducted properly, the condition of staining or the interval of sample preservation might have affected the results. In fact, the proportion of MMR-deficient cases (14.5%) in our cohort was higher than those in other previous studies (5–9%).^10,36,37^ Third, tissue samples used for immunohistochemical analysis included both biopsy and surgically resected specimens, and this might have affected the evaluation of molecular expression due to intratumoural heterogeneity. A previous study showed that PD-L1 positivity varied markedly within the tumour of head and neck squamous cell carcinoma,^38^ but little is known about the heterogeneity of molecular expression in GC. Since biopsy specimens are often used to evaluate tumour characteristics in clinical practice, further studies are required to investigate whether biopsy specimens show similar characteristics to surgically resected specimens in GC.

In conclusion, our results indicated that several clinicopathological characteristics showed a significant association with nivolumab responders and HPD patients. A combination of these factors might enable the identification of patients who will either benefit from or be harmed by nivolumab treatment for GC. This study focused only on relatively basic clinical and immunohistochemical characteristics, and did not examine other factors such as genomic alterations. Thus, these easily evaluated factors might be used as cost-effective screening tools that can be routinely used in clinical practice when administering nivolumab treatment to patients with GC.

## Supplementary information

Supplemental Figures

Supplemental Table

## Data Availability

All data and materials are available in this study.
