# Supplementary figures and images for: Multicentre biomarker cohort study on the efficacy of nivolumab treatment for gastric cancer
Source: Br J Cancer. 2020 Jul 3;123(6):965–72. doi: 10.1038/s41416-020-0975-7 (PMC7492241; doi:10.1038/s41416-020-0975-7)

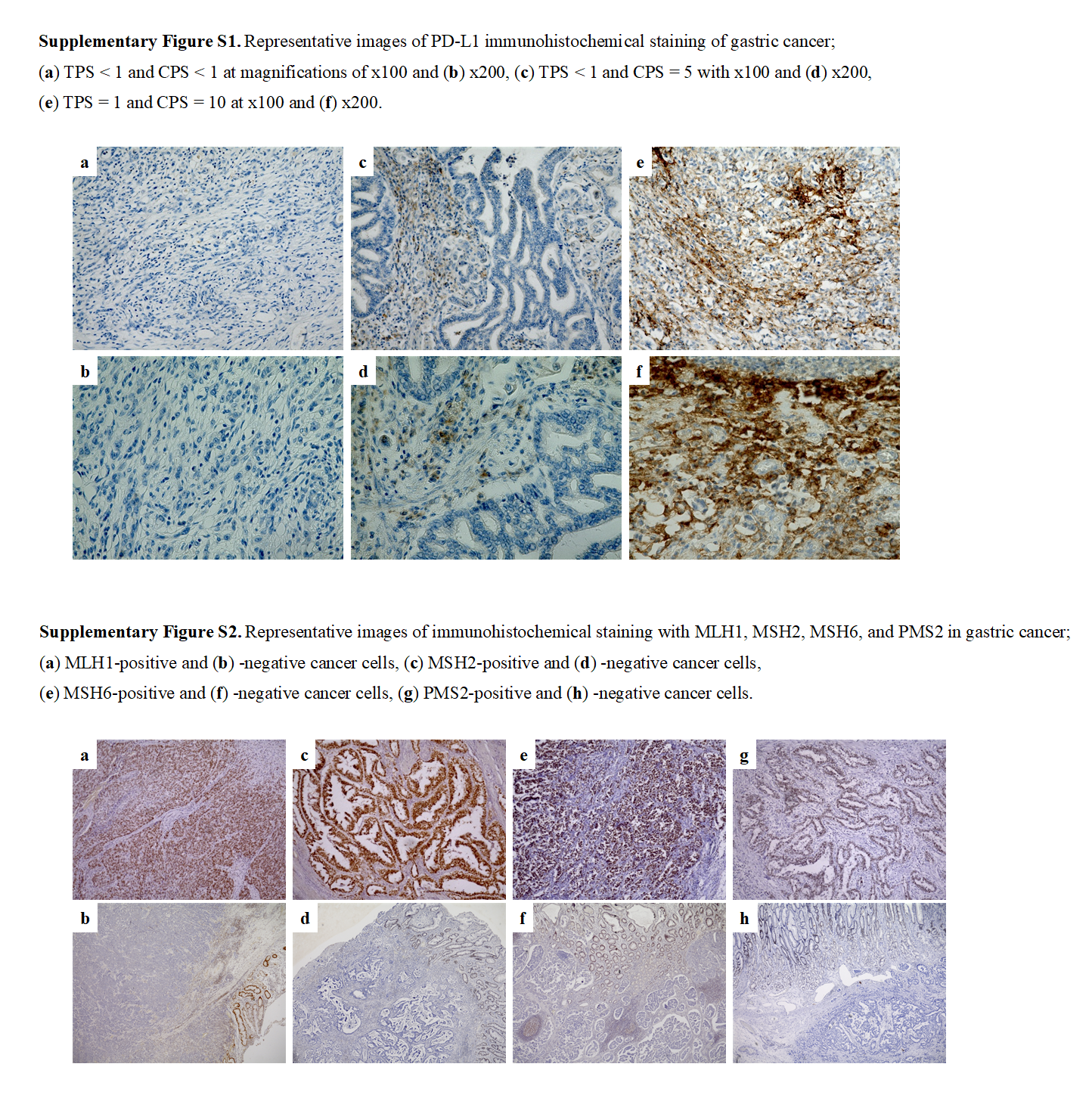

Supplement: Supplementary file 1 — Supplemental Figures [file 41416_2020_975_MOESM1_ESM.tif]
