# Supplementary material for: Multicentre biomarker cohort study on the efficacy of nivolumab treatment for gastric cancer
Source: Br J Cancer. 2020 Jul 3;123(6):965–72. doi: 10.1038/s41416-020-0975-7 (PMC7492241; doi:10.1038/s41416-020-0975-7)
Supplement: Supplementary file 2 — Supplemental Table [file 41416_2020_975_MOESM2_ESM.docx]

| Name of institution | Name of the ethics committee | Reference number |
| --- | --- | --- |
| Osaka University Hospital | Ethical Review Board of Osaka University Hospital | 17251 |
| Osaka Rosai Hospital | Osaka Rosai Hospital Institutional Review Board | 29-91 |
| Osaka International Cancer | Osaka International Cancer Institute Review Board | 1801199346-4 |
| Higashiosaka City Medical Center | Higashiosaka City Medical Center Institutional Review Board | 02-0401-A |
| Osaka General Medical Center | Osaka General Medical Center Institutional Review Board | 29-C0805 |
| National Hospital Organization Osaka National Hospital | National Hospital Organization Osaka National Hospital Institutional Review Board | 18047 |
| Ikeda Municipal Hospital | Ethics Committee of Ikeda Municipal Hospital | 3330 |
| Sakai City Medical Center | Sakai City Medical Center Institutional Review Board | 88 |
| Osaka Police Hospital | Osaka Police Hospital Institutional Review Board | 838 |
| Saito Yukoukai Hospital | Saito Yukoukai Hospital Institutional Review Board | 58 |
| Hyogo Nishinomiya Hospital | Hyogo Nishinomiya Hospital Ethics Committee | H29-43 |
| Rinku General Medical Center | Ethics Committee for Clinical Research, Rinku General Medical Center | 30-14 |
| Minoh City Hospital | Ethics Committee of Minoh City Hospital | H300404A |
| Kaizuka City Hospital | Kaizuka City Hospital Institutional Review Board | 172 |
| Toyonaka Municipal Hospital | Toyonaka Municipal Hospital Institutional Review Board | 2017-12-07 |
| Moriguchi Keijinkai Hospital | Moriguchi Keijinkai Hospital Institutional Review Board | 3004 |
| Kansai Rosai Hospital | Kansai Rosai Hospital Institutional Review Board | 20C022g |
| Itami City Hospital | Ethics Committee of Itami City Hospital | 307 |
| Yao Municipal Hospital | Ethics Committee of Yao Municipal Hospital | YMHH29-38 |
| Saiseikai Tondabayashi Hospital | Saiseikai Tondabayashi Hospital Institutional Review Board | 29-11-1 |
| Suita Municipal Hospital | Suita Municipal Hospital Institutional Review Board | 2018-ken1 |
| Osaka Saiseikai Senri Hospital | Osaka Saiseikai Senri Hospital Ethical Committee | 320110 (300504) |
| Nishinomiya Municipal Central Hospital | Nishinomiya Municipal Central Hospital Institutional Review Board | 461 |

**Supplemental Table.** Names of the ethics committees of all participating institutions and the reference numbers of this study
